# Athletic humans and horses: Comparative analysis of interleukin-6 (IL-6) and IL-6 receptor (IL-6R) expression in peripheral blood mononuclear cells in trained and untrained subjects at rest

**DOI:** 10.1186/1472-6793-11-3

**Published:** 2011-01-21

**Authors:** Stefano Capomaccio, Katia Cappelli, Giacomo Spinsanti, Marzia Mencarelli, Michela Muscettola, Michela Felicetti, Andrea Verini Supplizi, Marco Bonifazi

**Affiliations:** 1 Centro di Studio del Cavallo Sportivo, Dipartimento di Patologia, Diagnostica e Clinica Veterinaria, Università degli Studi di Perugia, Via San Costanzo 4, 06126 Perugia, Italia; 2 Dipartimento di Biologia Evolutiva, Università di Siena, Via Aldo Moro, I-53100 Siena, Italia; 3 Dipartimento di Fisiologia, Università di Siena, Via Aldo Moro, I-53100 Siena, Italia; 4 Dipartimento di Scienze Neurologiche, Neurochirurgiche e del Comportamento Via Aldo Moro, I-53100 Siena, Italia

## Abstract

**Background:**

Horses and humans share a natural proclivity for athletic performance. In this respect, horses can be considered a reference species in studies designed to optimize physical training and disease prevention. In both species, interleukin-6 (IL-6) plays a major role in regulating the inflammatory process induced during exercise as part of an integrated metabolic regulatory network. The aim of this study was to compare IL-6 and IL-6 receptor (IL-6R) mRNA expression in peripheral blood mononuclear cells (PBMCs) in trained and untrained humans and horses.

**Results:**

Nine highly trained male swimmers (training volume: 21.6 ± 1.7 h/wk in 10-12 sessions) were compared with two age-matched control groups represented by eight lightly trained runners (training volume: 6.4 ± 2.6 h/wk in 3-5 sessions) and nine untrained subjects. In addition, eight trained horses (training volume: 8.0 ± 2.1 h/wk in 3-4 sessions) were compared with eight age-matched sedentary mares. In humans, IL-6 mRNA levels in PBMCs determined by quantitative reverse transcription-polymerase chain reaction were significantly higher in highly trained subjects, whereas IL-6R expression did not differ among groups. In horses, transcripts of both IL-6 and IL-6R were significantly up-regulated in the trained group.

**Conclusions:**

Up-regulation of IL-6R expression in PBMCs in horses could reflect a mechanism that maintains an adequate anti-inflammatory environment at rest through ubiquitous production of anti-inflammatory cytokines throughout the body. These findings suggest that the system that controls the inflammatory response in horses is better adapted to respond to exercise than that in humans.

## Background

As species, humans and horses are closely linked, not only because of their historical and cultural backgrounds, but also because they share a natural aptitude for athletic performance. This similarity has prompted some researchers to consider the horse a reference species for comparative studies in human exercise physiology; conversely, knowledge gained from human medicine frequently represents a starting point for research on veterinary exercise medicine [1-4]. This topic is of interest for both species, as evidenced by the number of studies that have reported on efforts to identify genes involved in the response to moderate activity and/or strenuous exercise [5-10]. These previous studies have provided evidence that oxidative stress during exercise is a physiological event that is common among exercising mammals.

Recent years have seen an exponential increase in specific molecular information, opening new paths of knowledge and providing interesting results that could be used to optimize physical training and prevent diseases. In particular, studies on the endocrinology of exercise and training have demonstrated the existence of an integrated metabolic network involved in regulating hormones and cytokines [11]. Among the components of this hormone-regulatory network is the pleiotropic cytokine, interleukin-6 (IL-6), which modulates the function of immune cells in response to exercise and training, thereby playing a major role in the exercise-induced inflammatory process [12].

Strenuous, prolonged exercise induces an increase in pro-inflammatory cytokines such as tumor necrosis factor-α (TNF-α), and dramatically up-regulates the inflammation-responsive cytokine IL-6. This response is balanced by the release of cytokine inhibitors, such as IL-1 receptor antagonist (IL-1ra) and the anti-inflammatory cytokine IL-10 [13]. The effects of IL-6 are believed to play a dominant role in this context. According to Petersen and Pederson [14], IL-6 exerts anti-inflammatory effects by inducing the release of IL-1ra and IL-10. IL-6 also inhibits TNF-α production both in vitro and in animal studies [12,15]. On the basis of these observations, it has been proposed that exercise exerts a protective, long-term anti-inflammatory effect [14].

In trained horses, Donovan and colleagues [16] found a strong increase in IL-6 mRNA expression in leukocytes after acute exercise. In humans, IL-6 gene expression after acute exercise has been reported to increase in monocytes [17] or remain unchanged in peripheral blood mononuclear cells (PBMCs) [18,19] (Moldoveanu et al., 2000; Connolly et al., 2004). IL-6 mRNA levels in human skeletal muscle is markedly increased by exercise, and contracting muscle appears to be the primary contributor to the exercise-induced increase in circulating levels of IL-6 [12].

The evidence that baseline IL-6 plasma concentrations are affected by training is limited, and the results of such studies are often contradictory [11,12,20-23], partly due to differences in experimental design, tissues studied and techniques used. In at least one study, expression of IL-6 mRNA in human muscle at rest did not change in response to training [21].

IL-6 exerts its action via a specific IL-6 receptor (IL-6R). In response to physical training, basal IL-6R mRNA levels in skeletal muscle are increased, suggesting a sensitization of skeletal muscle to IL-6 at rest [24]. To our knowledge, it is not known if enhanced IL-6R expression following training also occurs in other tissues. In this regard, because anti-inflammatory cytokines such as Il-10 and Il-1ra are mainly produced by IL-6-stimulated macrophages, lymphocytes and monocytes [12], the role of circulating immunocytes in controlling the inflammatory response to exercise remains crucial.

In a recent case-control study, it was shown that glucocorticoid receptor alpha (GR-α) mRNA levels in PBMCs at rest were much lower in highly trained athletes than in sedentary subjects, suggesting that training may induce an adaptive mechanism that modulates the sensitivity of the target tissue [25]. By extension, determining the resting state of IL-6 and IL-6R mRNA expression in PBMCs of high-level athletes compared with lightly trained and sedentary subjects can be useful in understanding the modulation of IL-6 in the adaptive response of circulating immunocytes to exercise training. Hence, the aim of the present case-control study was to compare IL-6 and IL-6R mRNA expression in PBMCs in highly trained, lightly trained and untrained male humans and in trained and untrained horses.

## Results

### Evaluation of the stability of selected housekeeping genes

#### Humans

Two different analytical procedures were used to test the reliability of selected control genes, both of which produced results that were highly similar overall. The *geNorm *and *NormFinder *algorithms were applied to analyze the expression data for the housekeeping genes (HKGs) β-actin (ACTB), glyceraldehyde phosphate dehydrogenase (GAPDH), cyclophilin B/peptidylprolyl isomerase B (CYPB/PPIB), and hypoxanthine phosphoribosyltransferase 1 (HPRT1), both independently for each of the tested groups and on all the data considered collectively (Table 1). The results showed that GAPDH was always the least stable control; thus, its use to normalize quantitative reverse transcription-polymerase chain reaction (qRT-PCR) experiments on human PBMCs samples should preferably be avoided. The remaining three HKGs (ACTB, CYPB/PPIB, and HPRT1) shared very similar behaviors (Table 1), and were always classified among the first positions in the stability rankings produced by *geNorm *and *NormFinder*. Specifically, HPRT1 showed the best expression stability within each single group and was always identified as the optimal control gene in this context (Table 1); however, it exhibited greater variability in expression in the entire dataset (compared to CYPB/PPIB and ACTB), and was therefore classified as the third-best gene in the comprehensive analysis. ACTB and PPIB showed very similar M-values (calculated by *geNorm*) and SD-values (calculated by *NormFinder*), always occupying the second or third positions in the stability rankings. These are classified as the best pair of reference genes in the comprehensive analysis (Table 1).

**Table 1:** Rankings of the four tested control genes for human athletes produced by *geNorm *and *NormFinder *for each of the three groups of samples and for the overall analysis

	**Sedentary**		**Low-level athletes**		**High-level athletes**		**Total**	
	***geNorm*** **(M value)**	***NormFinder*** **(SD value)**	***geNorm*** **(M value)**	***NormFinder*** **(SD value)**	***geNorm*** **(M value)**	***NormFinder*** **(SD value)**	***geNorm*** **(M value)**	***NormFinder*** **(SD value)**
	
**1**	HPRT1/ACT-B^* ^ (0.28)	HPRT1 (0.22)	HPRT1/ACT-B^* ^ (0.24)	HPRT1 (0.12)	HPRT1/ACT-B^* ^ (0.31)	HPRT1 (0.15)	ACT-B/HCYPB^* ^ (0.47)	ACT-B (0.13)
**2**		HCYPB (0.31)		ACT-B (0.13)		ACT-B (0.16)		HCYPB (0.41)
**3**	HCYPB (0.40)	ACT-B (0.32)	HCYPB (0.30)	HCYPB (0.36)	HCYPB (0.37)	HCYPB (0.16)	HPRT1 (0.54)	HPRT1 (0.53)
**4**	GAPDH (0.53)	GAPDH (0.60)	GAPDH (0.41)	GAPDH (0.49)	GAPDH (0.44)	GAPDH (0.18)	GAPDH (0.65)	GAPDH (0.65)

*Denotes the two most stable genes that constitute a couple that cannot be further ranked (Vandesompele et al., 2002). M values (calculated according to the *geNorm *algorithmic procedures; Vandesompele et al., 2002) and SD values (calculated according to the *NormFinder *algorithmic procedures; Andersen et al., 2004) constitute different measures of the stability of gene expression across samples.

On the basis of the expression stability values collected in this study, an optimal normalization factor for qRT-PCR data could be calculated using the three most stable genes (ACTB, hCypB, and Hprt1), and excluding the lowest-scoring GAPDH.

#### Horses

A previous study employing *geNorm*, *NormFinder *and *BestKeeper *algorithms (Cappelli et al., 2008) indicated that succinate dehydrogenase complex subunit 1 (SDHA) and hypoxanthine phosphoribosyl transferase (HPRT) were the best housekeeping genes from among a group of nine potential genes tested. mRNA transcripts of these genes were used here to calculate an optimal normalization factor for qRT-PCR data.

### Differences in IL-6 and IL-6R mRNA expression

IL-6 mRNA was up-regulated in highly trained subjects in both species. In contrast, IL-6R was strongly induced by physical training in horses, but showed little change in response to training in humans.

**Figure 1:**
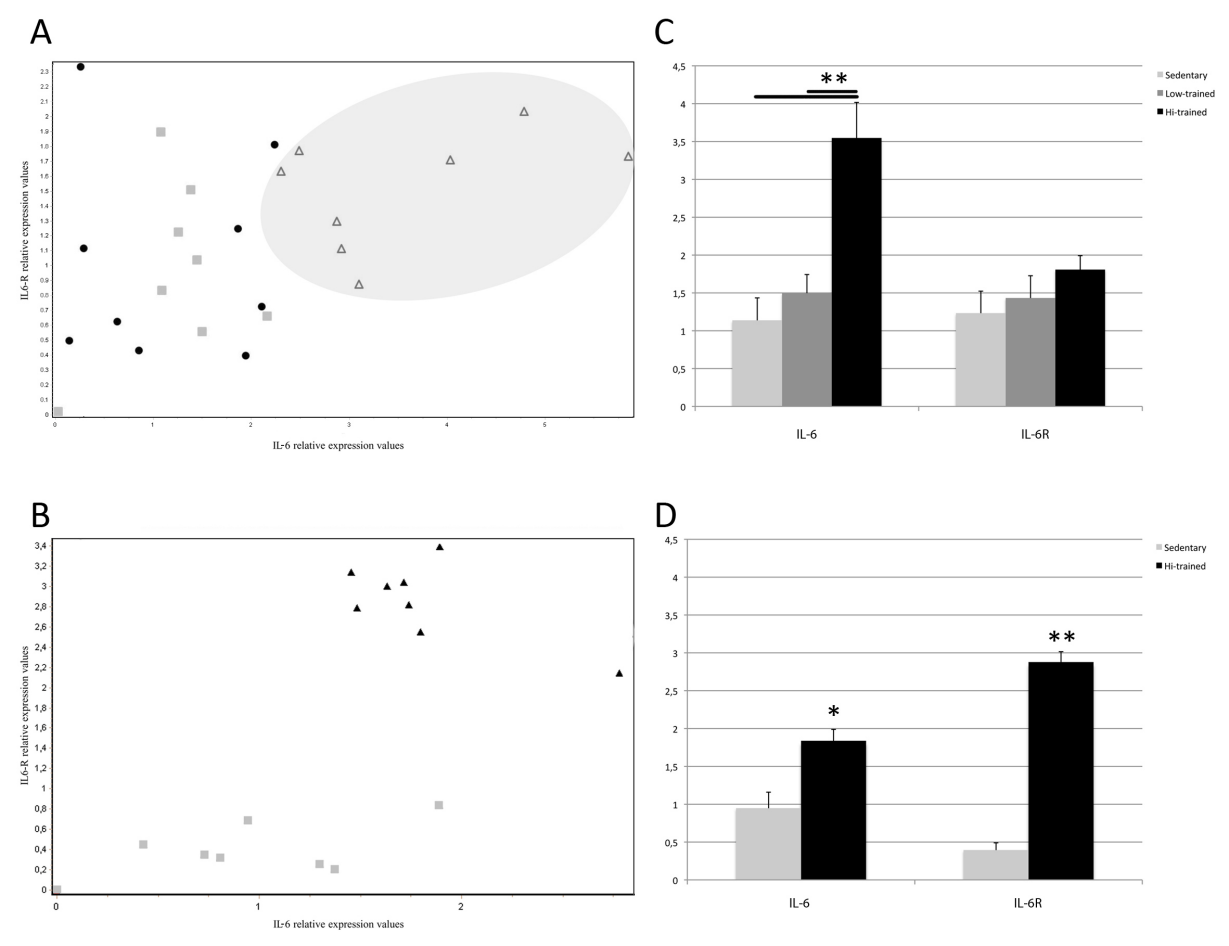
**IL-6 and IL-6R relative expression scatter plot**. (A-B) Scatter plot depicting the relative expression values of IL-6 (x-axis) and IL-6R (y-axis) among samples and groups according to Real-Time PCR data analysis described in the "Methods" chapter of the manuscript. A: Human subjects. Sedentary group, black dots; lightly trained athletes, gray squares; highly trained athletes, white triangles. The highly trained athletes group is encompassed by a grey ellipse. B: Horse subjects. Sedentary group, black triangles; highly athletic horses, grey squares. (C-D) Bar plots showing fold-differences in expression of IL-6 and IL-6R among groups (descriptive statistic). C: Human subjects. Sedentary group, grey bars; lightly trained athletes, dark grey bars; highly trained athletes, black bars. D: Horse subjects. Sedentary group, dark grey bars; highly athletic horses, black bars. Error bars indicate the standard error calculated using a 95% confidence interval. Relative expression values (fold change) are on a logarithm base 2 scale (*P < 0.05, **P < 0.001).

The scatter plot displayed in Figure 1A-B, which shows the correlation between IL-6 and IL-6R expression ratios for each of the individuals tested, clearly reveals the graphic separation of highly trained human athletes from other samples, mainly due to their higher levels of IL-6 expression (Figure 1A). It is also evident that lightly trained athlete- and sedentary-subject groups tended to overlap and were not significantly differentiated on the basis of changes in IL-6 or IL-6R expression. More evident is the separation within horses groups due to higher levels IL-6 and IL-6R expression in trained subjects (Figure 1B). This trend in both humans and horses is also evidenced by the reduced standard error in the bar plot of Figure 1C-D, which compares the relative expression of IL-6 and IL-6R across all groups.

The bar plot results presented in Figure 1C and 1D demonstrate the statistical significance of IL-6 over-expression in athletes compared with the other groups in both humans and horses, showing 2.451- and 2.358-fold inductions (logarithmic base 2) for highly trained human athletes versus sedentary and lightly trained level athletes, respectively, and a 0.948-fold induction (logarithmic base 2) for athletic compared with sedentary horses. On the other hand, the slight IL-6R induction in human athletes was not statistically significant, in contrast with the robust (2.505-fold, logarithmic base 2) and highly significant induction of IL-6R in athletic horses compared with sedentary horses.

## Discussion and Conclusions

Our results show the effect of high-level exercise training on the induction of the IL-6 and IL-6R genes in two species: *Homo sapiens *and *Equus caballus*. In humans, transcription of the IL-6 gene is clearly higher in highly trained subjects, whereas the expression of IL-6R does not differ significantly among the groups. In horses, both transcripts are significantly expressed at higher levels in athletic individuals.

Cytokines modulate immune cell function and migration, initiating and amplifying the acute-phase and stress responses; the local production of these molecules coordinates the function of innate and adaptive immune cells [18]. In this context, it is widely presumed that IL-6 could mediate the protective, long-term anti-inflammatory effects of exercise by orchestrating an anti-inflammatory reaction involving macrophages, lymphocytes and monocytes. In fact, IL-6 stimulates the production of cytokine inhibitors (IL-1ra) and anti-inflammatory cytokines (e.g., IL-10) in these immunocytes [12,14,26].

Endurance races in humans (i.e., marathons) and in horses (i.e., endurance races up to 160 km) have been shown to induce similar responses in both species [13]. After a marathon, there is a dramatic increase in IL-6 (128-fold) concomitant with an increase in the pro-inflammatory cytokines TNF-α (2.3-fold) and IL-1β (2.1-fold). This is balanced by the release of cytokine inhibitor IL-1ra (39-fold) and the anti-inflammatory cytokine IL-10 (28-fold), which restrict the magnitude and duration of the inflammatory response to exercise. In horses, it has been reported that strenuous, long-duration exercise triggers a systemic pro-inflammatory response similar to the events that occur during sepsis, with increased levels of circulating cytokines IL-6 (120-fold), TNF-α (3-fold) and IL-1β (4-fold) [16].

In humans, contracting muscle has been reported to contribute most of the circulating IL-6 elaborated in response to endurance exercise [12], whereas the main source of circulating IL-6 after exercise in horses remains to be determined. In untrained men, Connolly and colleagues [19] found no global increase in the PBMC expression of IL-6 mRNA after 30 minutes of exercise. They did, however, note a significant parallel increase in the expression of IL-1ra and IL-6R in PBMCs after 1 hour of recovery from exercise. Interestingly, the timing of this increase in the expression of both genes in PBMCs paralleled the increase in circulating levels of IL-1ra [19]. An increase in the expression of IL-1ra in PBMCs after exhaustive exercise was also found by Buttner et al. [27] in moderately trained students, and by Zieker et al. [28] in well-trained runners. In addition, it was recently shown that IL-1ra gene variants are associated with athletic status [29]. However, Rhind et al. [17] reported that physical exercise triggers increased expression of IL-1β, IL-1ra, IL-6 and TNF-α in human PBMCs, and showed that the intracellular up-regulation of inflammation-associated cytokines corresponded with increased serum concentrations. From these observations, the authors concluded that blood monocytes can serve as a source of circulating inflammatory cytokine production with exercise. The extremely fatiguing exercise regimen used by Rhind and colleagues [17], consisting of seven days of exhausting exercise, may have provided a stronger stimulus for monocyte activation, and thereby greater cytokine synthesis, compared with previous experiments. In addition, it has been reported that, in highly trained athletes, mononuclear cells appear to be chronically activated to spontaneously release IL-6, but have a decreased response to further stimulation [30]. Consistent with a possible training-induced sensitization of PBMC to release IL-6, we found higher levels of IL-6 mRNA in the PBMCs of athletes than in untrained subjects, both in humans and horses.

IL-6 exerts its action via IL-6R, which exists both in a soluble (sIL-6R) and a membrane-bound form. Complexation of IL-6 with its soluble receptor, in fact, increases the half-life of IL-6 and allows signaling to occur in tissues devoid of IL-6Ra process termed trans-signaling. Few studies (only in humans) have addressed the effect of exercise on sIL-6R levels and the findings are conflicting. An increase in sIL-6R after exercise has been reported in sedentary middle-aged men [31] and in endurance-trained cyclists [23]. However, it has also been reported that, after exercise, IL-6 mRNA expression in muscle increases while IL-6R plasma protein remains unaffected, suggesting that IL-6R production mainly serves a local role [24].

Exercise training could affect IL-6 and IL-6R expression, although few studies have addressed this question. In humans, the magnitude of exercise-induced IL-6 mRNA expression in skeletal muscle in untrained subjects was markedly reduced after 10 weeks of endurance training, whereas IL-6 expression at rest did not change in response to training [21]. In horses, a light 8-week training program did not affect IL-6 or IL-10, but did increase IL-1ra mRNA levels in circulating leukocytes [20]. It has been shown that exercise training increases basal levels of IL-6R mRNA in skeletal muscle. This increase in IL-6R gene expression could support the metabolic actions of IL-6 on skeletal muscle, suggesting that IL-6 target tissues can be sensitized at rest to IL-6 after a training period. The IL-6R mRNA response to exercise in humans, unlike that of IL-6, is not affected by training status or energy availability, and the factors that modulate IL-6R induction have not been clearly identified [24]. However, it has been reported that, in human skeletal muscle, increases in cortisol and IL-6 after exercise could play a role in exercise-induced IL-6R expression [24].

To our knowledge, whether enhanced IL-6R expression following training also occurs in other tissues and in other species has not been established. In the present study, IL-6R gene expression in PBMCs was found to be higher in endurance-trained athletic horses than in untrained subjects. Up-regulation of IL-6R expression in PBMCs in horses could reflect a mechanism to maintain an adequate anti-inflammatory environment at rest through ubiquitous production of anti-inflammatory cytokines throughout the body.

These results could be interpreted to mean that the system that controls the inflammatory response in horses is better adapted to respond to exercise than that in humans. Further investigation into these differences should provide insight into what types and level of effort are required by these species to reach top condition and peak performance. However, the study design (a case-control study) limits the generalizability of the results. Additional studies will be needed to establish if trained horses develop (or need to develop) stronger adaptation mechanisms capable of maintaining an anti-inflammatory body environment at rest.

## Methods

### Sample collection and RNA extraction

#### Humans

Nine highly trained male athletes (25.7 ± 3.5 years), members of the Italian National Swimming Team, were monitored during the 2006 training season. The subjects were middle-distance swimmers ranked in the top 10 in the 2006 world list in individual or relay events. During the season, they performed a mean of 21.6 ± 1.7 hours of training per week distributed in 10-12 sessions per week. Highly trained athletes provided venous blood samples at 08:00 hours under resting conditions (i.e., at least 24 hours after a training session). Three weeks after sampling, all swimmers participated in the European Swimming Championships (Budapest, 31 July to 6 August 2006); all were individual or relay medalists in their selected event, and five won gold medals.

For comparisons, eight male lightly trained athletes (29.4 ± 5.3 years) and nine male untrained subjects (29.0 ± 6.4 years) were enrolled in the study. Lightly trained athletes were recreational runners with comparable training and performance levels in endurance competitions. They performed a mean of 6.4 ± 2.6 hours of endurance training per week, according to an individual program, distributed in 3-5 sessions per week. The untrained subjects were chosen from among sedentary individuals not engaged in any form of training. Both lightly trained and untrained groups provided a venous blood sample at 08:00 hours under resting conditions (i.e., at least 24 hours after a training session in the case of lightly trained athletes). All samples were collected between the last week of June and the first week of July, 2006. Each subject completed an epidemiological-anamnestic questionnaire that included training information, and underwent a medical examination to exclude any disease, fever or medical treatment that could affect the immune system.

The methods used in this study were in accordance with the Helsinki Declaration of 1975 as revised in 1996. All eligible participants were enrolled consecutively after obtaining written informed consent.

PBMCs were isolated by the Ficoll-Hypaque method (GE Healthcare, Pollards Wood, United Kingdom) immediately after collection of blood. Total RNA was extracted from 1 × 10^7 ^PBMCs using the RNeasy mini kit (Qiagen, Hilden, Germany) and resuspended in 50 μl RNase-free water. RNA integrity was assessed by electrophoresing on 1.6% (w/v) agarose gels (Amersham Pharmacia Biotech AB, Uppsala, Sweden), and RNA concentration was determined spectrophotometrically.

#### Horses

Eight trained horses (6.8 ± 2.44 years, six males and two females) were monitored during the 2007 training season. The subjects were chosen from among the participants in national and international endurance races (90-120 km). During the season, they performed a mean of 8 ± 2.1 hours of training per week distributed in 3-4 sessions per week.

Eight age-matched untrained horses were chosen from among sedentary mares. Venous blood samples were collected from both groups under resting conditions (i.e., at least 24 hours after a training session). All samples were collected between June and July, 2006.

Each horse owner completed an epidemiological-anamnestic questionnaire that included training information, and horses underwent a medical examination to exclude any disease, fever or medical treatment that could affect the immune system.

The animals involved in this study were treated following standard procedures to ensure animal welfare, in agreement with the horse owners, veterinarian and the official veterinary commission.

Total RNA was extracted using the Aurum Total RNA Fatty and Fibrous Tissue kit (Bio-Rad, Hercules, CA, USA) according to the manufacturer's instructions. Genomic DNA was eliminated by DNase treatment using reagents supplied with the kit. Extracted RNA was quantified using the Quant-It RNA assay (Invitrogen, Dorset, United Kingdom) in a VersaFluor fluorometer (Bio-Rad), and checked for integrity by denaturing agarose gel electrophoresis with ethidium bromide staining. Successful removal of DNA contaminants was tested by determining the absence of PCR amplification of the *MC1R *gene (GenBank accession number X98012, primers from Rieder and colleagues [32]).

### Reverse transcription

Human and horse total RNA (500 ng) was reverse transcribed using random hexamer primers and Superscript III Reverse Transcriptase (Invitrogen) according to the manufacturer's specifications. *Equus caballus *and *Homo sapiens *ACTB mRNA was amplified by PCR from each cDNA to verify successful reverse transcription.

### Design of qRT-PCR primers

Real-time PCR primers used to amplify two human housekeeping genes (ACTB and GAPDH) and the gene of interest, IL-6R, were taken from previous papers [33]. Real-time PCR primers for the remaining two human housekeeping genes (HPRT1 and CYPB/PPIB) and human IL-6 were designed based on the nucleotide sequences available in GenBank (Additional File 1), as were primers utilized to amplify horse genes (Additional File 2). Beacon Designer 7.06 (Premier Biosoft International) was used to design primers, giving special attention to primer length, annealing temperature, base composition and 3'-end stability. DNA polymerization efficiency was optimized by selecting amplicon lengths ranging from 69 to 174 bp (Additional File 1) for human genes and from 68 to 138 bp (Additional File 2) for horse genes. Primer sequences were analyzed using Mfold [34] to ensure that the chosen sequences did not coincide with regions of template secondary structure and, where possible, were located on different exons or on exon-exon junctions. Specificity of amplification was confirmed by sequencing.

For each pair of primers, a preliminary real-time assay was performed to check for amplification of non-specific products or primer-dimer artifacts (no double peak in melt-curve analysis), and slope values and the correlation coefficients (R2) were determined to assess the efficiency (E) of qRT-PCR. Human genes were amplified on an iQ5 machine (Bio-Rad) using serial 1:3 dilutions of template cDNA, and horse genes were amplified on an MX3000P instrument (Stratagene) using 4-fold serial dilutions of template cDNA (Additional file 1 and Additional file 2). Products were run on 2% agarose gels to check for size specificity and were subsequently sequenced.

### Quantitative real-time PCR

#### Humans

Real-time amplifications were run in triplicate on 96-wells reaction plates using SYBR Green detection chemistry and an iQ5 thermocycler (Bio-Rad). Reactions were prepared in a total volume of 20 μl containing 1 μl cDNA, 0.8 μl of each 10 μM primer (300 mM each), 10 μl of iQ SYB Green Supermix (Bio-Rad) and 8 μl RNase/DNase-free sterile water (Qiagen). Blank controls were run in triplicate for each master mix. The following cycling conditions were used: 95°C for 1 minute (initial template denaturation), followed by 40 cycles of denaturation at 95°C for 10 seconds, and combined primer annealing/elongation at 60°C for 30 seconds, as described previously [35]. Thermocycling was followed by a melting-curve analysis, ranging from 56°C to 95°C, with temperature increasing in 0.5°C steps every 10 seconds.

Baseline and threshold values were automatically determined for all plates using Bio-Rad iQ5 Software 2.0. Raw Ct values were imported into GenEx Pro (version 4.3.5) and analyzed.

#### Horses

Five microliters of cDNA template (previously diluted 1:10) were added to the FastStart SYBR Green Master mix (Roche Applied Science, Penzberg, Germany) containing an internal ROX fluorochrome standard. PCR reactions were performed in a volume of 25 μl on an Mx3000P instrument (Stratagene, La Jolla CA, USA). PCR conditions were the same for all primer pairs: initial denaturation at 95°C for 10 minutes followed by 40 cycles of denaturation at 95°C for 30 seconds, annealing at 58°C for 30 seconds and extension at 72°C for 30 seconds. Fluorescence data were collected at the end of the extension step. Following cycling, the melting curve was determined in the range 58°C-95°C, with a temperature increment of 0.01°C/s. Each reaction was run in triplicate with appropriate negative controls.

Baseline and threshold values were automatically determined for all plates using the MxPro 3000P. Raw Ct values were imported into GenEx Pro and analyzed.

### Reliability of housekeeping genes

The stability of housekeeping gene expression was tested in both species using *geNorm *and the *NormFinder *visual basic applications, two of the most diffused algorithms for the statistical determination of suitable reference genes in a qRT-PCR experiment.

*geNorm *compares the variations in all candidate gene expression ratios and sequentially eliminates those that show the highest variation. Selected HKGs are ranked according to the determined control gene-stability measure (M, average pair-wise variation of a particular gene with all other control genes), from the most stable (lowest M values) to the least stable (highest M values). The last pair of remaining candidate genes that cannot be further ranked is recommended as the optimum pair of reference genes.

*NormFinder *compares the variation of every gene, identifying the optimum reference gene. The most obvious advantage of *NormFinder *is that it examines the stability of each single candidate gene independently and not in relation to the other genes, as *geNorm *does [36]. This is important given uncertainties regarding potential co-regulation of target genes. *NormFinder *can also analyze samples belonging to different groups and calculate both intra-group variation (stability of gene expression within groups) and intra-group variability (variations in gene expression between groups). Based on these results, *NormFinder *also recommends an optimal pair of reference genes. The pair of genes selected may exhibit compensatory expression, such that one reference gene that is slightly over-expressed and one that is slightly over-expressed in the same group of samples are recommended as the reference genes. Selection of genes that may compensate for each other's fluctuations is mainly helpful in situations where none of the candidate reference gene transcripts is stably expressed.

### Analysis of real-time PCR data

The raw Ct values determined during qRT-PCR experiments were subdivided into three groups for humans (sedentary, n = 9; lightly trained athletes, n = 8; highly trained athletes, n = 8) and two groups for horses (athletic, n = 8; sedentary, n = 8), and then analyzed using GenEx Pro software. During the pre-processing phase, data were corrected for PCR efficiencies (Additional file 1 and Additional file 2) and the three technical repeats were averaged. The selected reference genes, ACTB, CYPB/PPIB and HPRT1 for humans, and HPRT1 and SDHA for horses [37], were subsequently used to normalize Ct values, and quantities were calculated relative to the maximum Ct value. Because our interest was in fold changes in gene expression between groups, we ultimately converted quantities to a logarithmic scale using a log base 2 conversion, which also allowed us to test the normal distribution of values.

For descriptive statistics, groups were tested for parametric data distribution using the Kolmogorov-Smirnov (KS) Test. Changes in the relative expression of IL-6 and IL-6R between groups were calculated using a two-tail unpaired t-Test where the data were normally distributed, and a Mann-Whitney Test for unpaired data where the data were non-normally distributed. Independent comparisons were performed between sedentary and lightly trained athletes, between sedentary and highly trained athletes, between lightly and highly trained athletes, and between athletic and sedentary horses. Statistical analyses were performed using GenEx Pro software.

## Authors' contributions

SC, KC and GS performed all the experimental procedures, data analysis and drafted the manuscript. MrM, MM and MF participated in sample collection, RNA extraction and analysis. AVS and MB conceived of the project, participated in study development and coordination, and co-wrote the manuscript. All authors read and approved the final manuscript.

## Supplementary Material

Additional file 1 **Table S1a. qRT-PCR primer pairs and details of amplicons (*Homo sapiens*)**. † refers to RT-PCR primers taken from Vandesompele et al. (2002); ‡ refers to primers taken from Koyama et al (2008). Click here for file

Additional file 2 **Table S1b. qRT-PCR primer pairs and details of amplicons (*Equus caballus*)**. Click here for file
