# Supplementary material for: Athletic humans and horses: Comparative analysis of interleukin-6 (IL-6) and IL-6 receptor (IL-6R) expression in peripheral blood mononuclear cells in trained and untrained subjects at rest
Source: BMC Physiol. 2011 Jan 21;11:3. doi: 10.1186/1472-6793-11-3 (PMC3036646; doi:10.1186/1472-6793-11-3)
Supplement: Additional file 1 — Table S1a. qRT-PCR primer pairs and details of amplicons (Homo sapiens). † refers to RT-PCR primers taken from Vandesompele et al. (2002); ‡ refers to primers taken from Koyama et al (2008). [file 1472-6793-11-3-S1.DOC]

**Table S1a.** qRT-PCR primer pairs and amplicons details (*Homo sapiens*).

| **Gene** | Acc. Number | Forward Primer Sequence  [5’→3’] | Position in cDNA | Reverse Primer Sequence  [5’→3’] | Position in cDNA | Amplicon Length | E% | R^2^ |
| --- | --- | --- | --- | --- | --- | --- | --- | --- |
| **ACT-B^†^** | **-** | CTGGAACGGTGAAGGTGACA | 6^th^ | AAGGGACTTCCTGTAACAATGCA | 6^th^ | 140 bp | 96.6 | 0.99 |
| **GAPDH^†^** | **-** | TGCACCACCAACTGCTTAGC | 7^th^ | GGCATGGACTGTGGTCATGAG | 7^th^/8^th^ | 87 bp | 91.8 | 0.998 |
| **HPRT1** | **NM_000194** | AGATGGTCAAGGTCGCAAG | 6^th^ | GTATTCATTATAGTCAAGGGCATATC | 8^th^ | 128 bp | 104.2 | 0.995 |
| **HCYPB** | **M60857** | CCAACGCAGGCAAAGACACCAA | 4^th^ | GCTCTCCACCTTCCGCACCA | 5^th^ | 131 bp | 91.2 | 0.995 |
| **IL6** | **NM_000600** | CCACTCACCTCTTCAGAACGAAT | 2^nd^ | TTGGAAGCATCCATCTTTTTCA | 3^rd^ | 174 bp | 99.7 | 0.992 |
| **IL6-R**^‡^ | **-** | GAGGGCTTCTGCCATTTCTGAG | 3’ UTR | CCAGGTTCAGCTGACAACAAACA | 3’UTR | 69 bp | 101.2 | 0.996 |

**^†^** refers to RT-PCR primers taken from Vandesompele et al. (2002); ^‡^ refers to primers taken from Koyama et al (2008).
