# Supplementary material for: Athletic humans and horses: Comparative analysis of interleukin-6 (IL-6) and IL-6 receptor (IL-6R) expression in peripheral blood mononuclear cells in trained and untrained subjects at rest
Source: BMC Physiol. 2011 Jan 21;11:3. doi: 10.1186/1472-6793-11-3 (PMC3036646; doi:10.1186/1472-6793-11-3)
Supplement: Additional file 2 — Table S1b. qRT-PCR primer pairs and details of amplicons (Equus caballus). [file 1472-6793-11-3-S2.DOC]

**Table S1b.** qRT-PCR primer pairs and amplicons details (*Equus caballus*).

| **Gene** | Acc. Number | Forward Primer Sequence  [5’→3’] | Position in cDNA | Reverse Primer Sequence  [5’→3’] | Position in cDNA | Amplicon Length | E% | R^2^ |
| --- | --- | --- | --- | --- | --- | --- | --- | --- |
| **IL6** | **AF041975** | TCAAGGGTGAAAAGGAAAACATC | 4/5^th^ | GGTGGTTACTTCTGGATTCTTC | 6^th^ | 98 bp | 100.8 | 0.998 |
| **IL6-R** | **XM_001494033** | CCTGTGGAGCGAGTGGAG | 7/8^th^ | ATTAGTAGTAGATGCCTGTGTGG | 7^th^/8^th^ | 109 bp | 97.8 | 0.999 |
| **HPRT1** | **AY372182** | AATTATGGACAGGACTGAACGG | 2^nd^/3^rd^ | ATAATCCAGCAGGTCAGCAAAG | 8^th^ | 121 bp | 93.2 | 1.000 |
| **SDHA** | **DQ402987** | GAGGAATGGTCTGGAATACTG | 14^th^ | GCCTCTGCTCCATAAATCG | 5^th^ | 91 bp | 96.0 | 0.999 |
